# Pleiotropic Effects of a Methyl Donor Diet in a Novel Animal Model

**DOI:** 10.1371/journal.pone.0104942

**Published:** 2014-08-14

**Authors:** Kimberly R. Shorter, Vanessa Anderson, Patricia Cakora, Amy Owen, Keswick Lo, Janet Crossland, April C. H. South, Michael R. Felder, Paul B. Vrana

**Affiliations:** 1 *Peromyscus* Genetic Stock Center University of South Carolina, Columbia, South Carolina, United States of America; 2 Dept. Biological Sciences, University of South Carolina, Columbia, South Carolina, United States of America; Pennington Biomedical Research Center/LSU, United States of America

## Abstract

Folate and other methyl-donor pathway components are widely supplemented due to their ability to prevent prenatal neural tube defects. Several lines of evidence suggest that these supplements act through epigenetic mechanisms (e.g. altering DNA methylation). Primary among these are the experiments on the mouse viable yellow allele of the agouti locus (A^vy^). In the A^vy^ allele, an Intracisternal A-particle retroelement has inserted into the genome adjacent to the agouti gene and is preferentially methylated. To further test these effects, we tested the same diet used in the A^vy^ studies on wild-derived *Peromyscus maniculatus*, a native North American rodent. We collected tissues from neonatal offspring whose parents were fed the high-methyl donor diet as well as controls. In addition, we assayed coat-color of a natural variant (wide-band agouti = A^Nb^) that overexpresses agouti as a phenotypic biomarker. Our data indicate that these dietary components affected agouti protein production, despite the lack of a retroelement at this locus. Surprisingly, the methyl-donor diet was associated with defects (e.g. ovarian cysts, cataracts) and increased mortality. We also assessed the effects of the diet on behavior: We scored animals in open field and social interaction tests. We observed significant increases in female repetitive behaviors. Thus these data add to a growing number of studies that suggest that these ubiquitously added nutrients may be a human health concern.

## Introduction

Folic acid and related B vitamins are widely supplemented in the US and western countries due to their ability to prevent neural tube defects such as spina bifida [1]. This consumption has increased over the last decade, due not only to direct supplementation (i.e. vitamin tablets/capsules) but also to enrichment of grains [2], [3], and addition to other products such as energy drinks e.g. (http://www.5hourenergy.com/QandA.asp).

While it is clear these compounds have beneficial effects, the underlying mechanisms are unknown. These molecules contribute to the 1-carbon/methyl donor pathway. This pathway contributes to many biological processes. Notably, these components are involved in production of SAM (S-Adenosyl Methionine), which is the ultimate donor responsible for adding methyl groups to proteins and nucleic acids. This and other data suggests that these nutrients act through epigenetic mechanisms, as methylation of DNA and histone amino acid residues are known to mediate epigenetic effects [4], [5].

These data include experiments on the viable yellow allele of the agouti locus (A^vy^) in the lab mouse. In the A^vy^ allele, an Intracisternal A-particle (IAP) class retroelement has inserted into the genome adjacent to the Agouti (a) gene [6]. The strength of the IAP promoter results in constitutive expression of the agouti locus. Thus, the A^vy^ allele results in hair that is all yellow (as opposed to hairs having regions of both black and yellow) as well as obesity and tumor predisposition. Maternal consumption of a diet high in components of the 1-carbon/methyl donor pathway restores A^vy^ animals to a wild-type appearance, presumably due to the observed increased DNA methylation of IAP promoter [4], [7], [8]. A similar IAP insertion at the axin locus (Axin^Fu^ allele) is similarly affected by the diet [9].

Few such studies have been done on natural variants or examination of other potential effects of such a diet. *Peromyscus* are wild-derived North American rodents and thus represent natural populations/genomes in ways that more widely used models do not [10]. *Peromyscus* have proven useful for evaluating the impacts of environmental factors. We therefore tested the (1X; Table 1) diet originally used in the A^vy^ studies on *P. maniculatus*. We employed a naturally occurring variant termed wide-band agouti (A^Nb^) as a biomarker for the effects of the diet [10], [11], [12]. The A^Nb^ allele is otherwise on a BW (http://stkctr.biol.sc.edu/wild-stock/p_manicu_bw.html) genetic background, a *P. maniculatus* stock whose genome has recently been sequenced (http://www.ncbi.nlm.nih.gov/assembly/84591/) and mapped [13]. Effects of the diet on the A^Nb^ animals would suggest general effects of the diet, as there is no evidence for a retroelement in this allele [14].

**Table 1:** Comparison of differing components in Harlan-Teklad (http://www.harlan.com/) Standard rodent (8604) vs. Methyl-Donor (7517) diet (g/kg of chow).

	Standard (8604)	Methyl Donor (7517)
Betaine	0	5
Choline	2.53	7.97
Folic Acid	0.0027	0.0043
Vitamin B12	0.051	0.53

BW animals have a tendency towards repetitive behaviors (e.g. jumps, backflips), and thus have been used as a model for Autism Spectrum (ASD) and Obsessive-Compulsive disorders (OCD) [15], [16], [17], [18], [19], [20]. We therefore wished to assess whether the diet overtly affected behavior in addition to potential effects on the A^Nb^ allele. These studies provide novel evidence of deleterious effects of large doses of these compounds typically considered therapeutic or preventive to disease.

## Methods

### Ethics Statement

All procedures were approved by the University of South Carolina Institutional Animal Care and Use Committee (IACUC; protocol #1809-100340-061011).

### Animal Husbandry & Mating Schemes

Animals were taken from the stocks maintained at the *Peromyscus* Genetic Stock Center (http://stkctr.biol.sc.edu/). Animals were kept on a 16∶8 hour light-dark cycle and were given food and water ad libitum. Matings of BW female×A^Nb^ male were established and maintained on either the methyl donor diet (Table 1) or normal rodent chow (i.e. controls). Offspring were weaned at approximately 25 days of age and maintained on the methyl donor diet or normal rodent chow until reaching six months of age (to obviate any concerns about maturity of coat-color; note that these animals live >4 yrs). Other animals were sacrificed at birth for future nucleic acid analyses; additional tissues from both ages are available to interested investigators.

### Behavioral Testing

Offspring of the BW female×A^Nb^ male matings were evaluated in Open Field and Social Interaction Tests at 4–6 months of age, as previously described [20]. We tested 62 experimental animals (39 ♀ & 23 ♂) and 30 controls (12 ♀ & 18 ♂). Briefly, these tests consisted of first placing a single animal into a standard rat (10.25″W×19″L×8″H) cage with aspen shavings and ventilated transparent cover. After five minutes of observation, we introduced a novel animal of the same sex and species. The subsequent five minute period constituted the social interaction test. The novel animal’s tail was marked with a non-toxic marker to distinguish it from the animal being tested. The cage was cleaned between each animal tested (including replacement of bedding).

All behaviors were recorded with a digital camcorder. We used the Noldus Observer XT software (http://www.noldus.com/) to score behaviors from the video data. For the open field test, we scored the following behaviors: burrowing, freezing, jumping, back-flipping, running in circles, and grooming. Based on these videos, we considered straight vertical jumping, back-flipping, and running circles as repetitive behaviors.

For the social interaction test videos, we scored the same behaviors as in the open field test with the addition of social and aggressive behaviors. General social behaviors included sniffing, following, and allogrooming. Aggressive behaviors included biting, chasing, boxing, and mounting.

All behaviors were scored by incidence; we assessed behavior type at five second intervals throughout the video. Three people scored each video; overall inter-rater reliability was at least 80 percent. At least two scorers were blind to the diet of the animals being scored. When specific behavioral assessments disagreed, we alternated accepting the assessment of the three scorers. The data collected by scoring videos were graphed with Microsoft Excel. Behaviors are reported as percentage of incidence of behavior. Statistics were calculated using the Minitab and SPSS software packages. Note that we used Kruskal–Wallis one-way analysis of variance in cases where there was clearly a non-normal distribution.

### Tissue Analyses

After behavioral testing, animals were euthanized via CO_2_ chamber. Whole pelts were taken in order to analyze coat color differences. Tissues (skin sample, brain, and liver) were obtained and flash frozen in liquid nitrogen.

### Measurement of Agouti (Yellow) Band Lengths

Hair tufts were pulled from the dorsal midline behind the ears from each pelt. Tufts of hair were placed on a microscope beside a micrometer and pictures were taken using a light microscope/digital camera combination. Agouti (yellow) band lengths in the hair were measured in millimeters (mm). We assessed 67 experimental animals (40 ♀ & 27 ♂) and 30 controls (12 ♀ & 18 ♂).

## Results

### Methyl Diet Affects Coat Color & Body Weight

**Figure 1:**
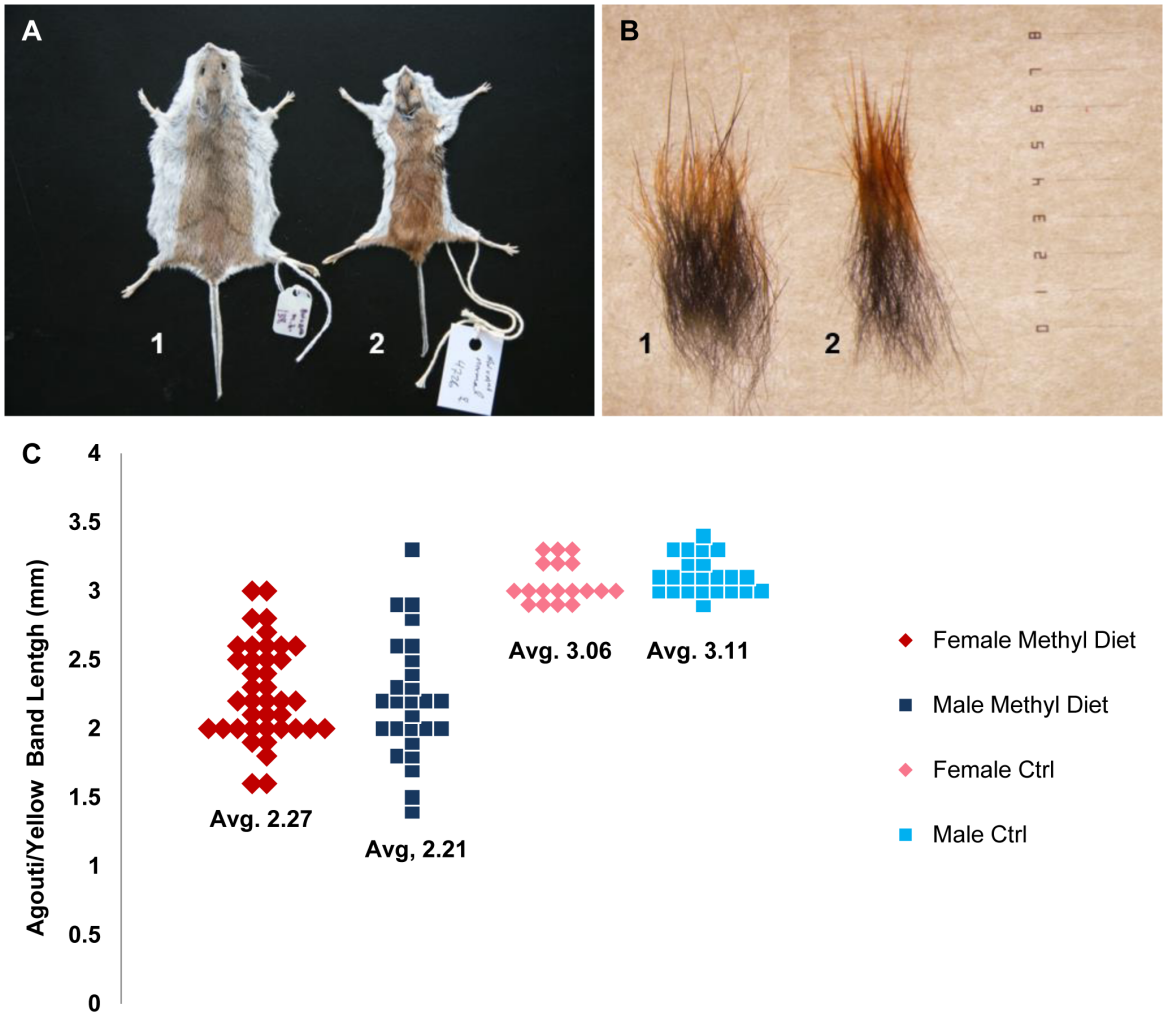
Effects of methyl-donor diet on coat-color/pattern. (A) Whole pelts and (B) corresponding hair tufts from representative six-month old female A^Nb^ methyl diet (#1) and control diet (#2) animals. Note the visible differences in yellow band length in hair tufts and size. (C) Distribution of yellow band lengths (in mm) in tufts of hair. T test was used to determine significance between methyl diet animals and control animals: t(107) = 15.9, p<0.005, d = 2.2. The calculated Cohen’s D value of 2.2 indicates a large treatment effect.

Matings were established to obtain offspring heterozygous for the dominant A^Nb^ allele. As this allele results in higher expression of agouti, heterozygotes exhibit a longer yellow band of hair and thus overall lighter appearance. A number of animals raised on the methyl-donor diet exhibited visibly darker coats than the controls (Fig. 1A).

To quantify these changes, we prepared pelts and measured the yellow (agouti) band length on the dorsal midline from 67 methyl diet animals (40♀, 27♂) and 41 controls (18♀, 23♂; Fig. 1B). These data revealed that while the control A^Nb^ animals had yellow band lengths tightly clustered around 3.1 mm, the treatment group had a broader distribution with an average yellow band length of 2.21 mm (Fig. 1C). These differences were deemed significant by T-test (p<0.005).

**Figure 2:**
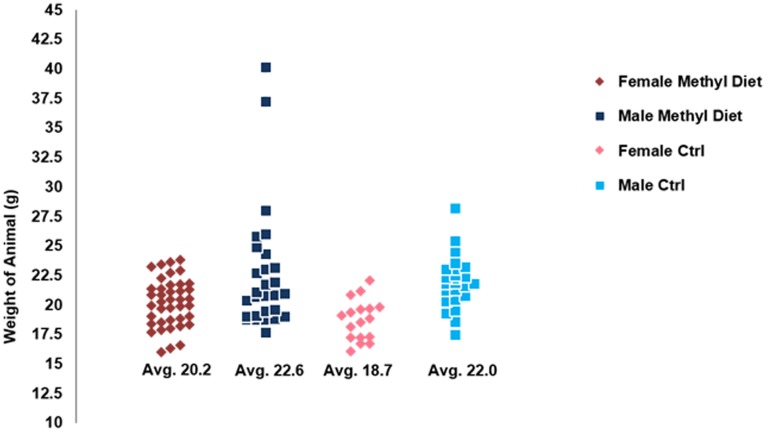
Weight distributions of methyl-diet vs control diet animals. We weighed 68 experimental animals (40 ♀ & 28 ♂) and 40 controls (12 ♀ & 18 ♂) at six months of age. The difference between female experimental & female control (ctrl) was significant (p<0.05; t-test), male averages were not significant. However, there were two methyl-diet males that were much larger than the control population.

A number of the methyl diet A^Nb^ animals appeared visibly larger than the controls. We therefore weighed the animals at the time of sacrifice (Fig. 2). Female methyl diet animals averaged 20.2 g compared to 18.7 g for control females; this shift was significant (p<0.05; t-test). Despite the presence of two much larger animals, the male methyl diet average (22.6 g) was essentially the same as the control average (22.0 g).

### Abnormalities & Mortality

Unexpectedly, we noted that a number of methyl-donor animals died between weaning and adult assessments of coat-color and behavior (4–6 months). While mortality was especially pronounced in males (p<0.001; Table 2), it was also significant in females (p = 0.005). Note that there was no mortality in control animals over this time period (*P. maniculatus* live 4–5 years in captivity).

**Table 2:** Mortality & abnormalities observed in methyl vs. control diet animals.

	Methyl Diet						Control Diet		
	♀ %	p value	♂ %	p value	% Litters	p value	♀ %	♂%	% Litters
Mortality	7.8	p = 0.005	22.2	p<0.001	47.1	p<0.001	0	0	0
Abnormalities:	10.6	p≤0.0025	32.1	p≤0.001	58.8	p≤0.001	0	0	0
Ovarian Cyst	6.4	N/A	N/A	N/A	17.6	N/A	0	0	0
Asym. Testes	N/A	N/A	10.7	N/A	17.6	N/A	0	0	0
Cataracts	2.1	N/A	7.1	N/A	11.8	N/A	0	0	0
Enlarged Liver	0	N/A	7.1	N/A	11.8	N/A	0	0	0
Other	2.1	N/A	10.7	N/A	23.5	N/A	0	0	0

**Figure 3:**
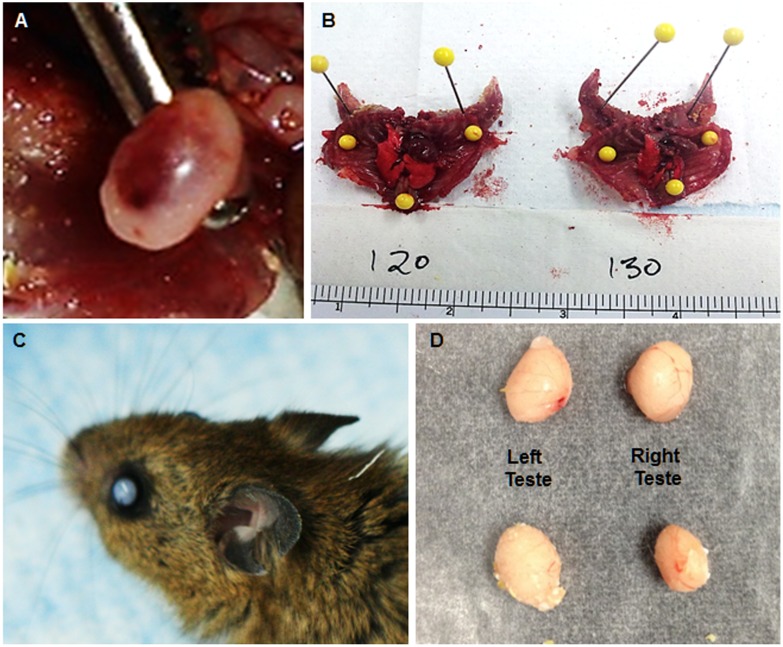
Representative abnormalities observed in methyl diet animals. (A) Hemorrhagic ovarian cyst in a methyl diet female. (B) Normal diet animal’s ribcage, heart, and lungs (left) compared to one methyl diet animal’s ribcage, heart and lungs; note abnormalities in size and shape of lungs and heart. (C) Cataracts were visible in the left eye of some animals. (D) Left and right testes from a control diet male (top) and a methyl diet male (bottom). Chi squared tests suggest significant size differences between right and left testes in these three methyl diet males.

When we took tissues from sacrificed animals for nucleic acid analyses, we noted a number of abnormalities in methyl diet animals not present in controls (Table 2). Again, the number was higher in methyl diet males (9 of 28 methyl diet males had at least one abnormality; p<0.005), but also significant in females (5 of 40 methyl diet females had at least one abnormality; p<0.01). These apparent defects (Table 2) were varied, and showed no effect of litter (i.e. were randomly distributed between the litters). They included ovarian cysts (Fig. 3A), size/consistency differences in ribcage, heart, and lungs (Fig. 3B), cataracts (Fig. 3C) and asymmetrical testes (Fig. 3D, E). In addition, we noted consistency differences in other organs (e.g. brain).

### Methyl Diet Affects Behavior

Animals still alive at six months were subjected to a simple open-field test and social interaction test, as described [20]. Major categories scored included repetitive behaviors (jumping, backflips, circle running) and general social behaviors (sniffing, following, allogrooming). We also assessed aggressive behaviors, including biting, boxing, mounting, and chasing.

**Figure 4:**
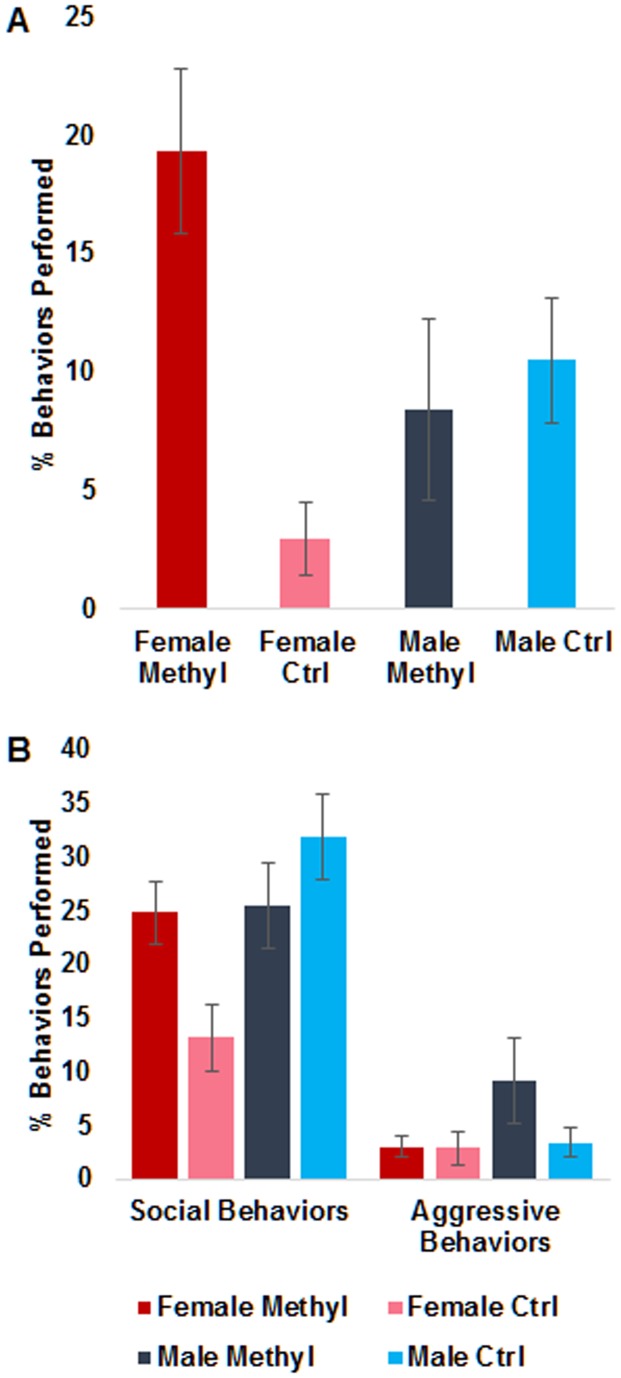
Effects of methyl-donor diet on behavior. (A) Repetitive behaviors in each group tested. Repetitive behaviors included jumping, back-flipping, and running in circles. Female methyl diet animals performed significantly higher numbers of repetitive behaviors than control diet females (p<0.01, Kruskal-Wallis test). (B) Social behaviors and aggressive behaviors for each group tested. Social behaviors included sniffing, following, and allogrooming. Female methyl diet animals were, on average, less social, but this was statistically insignificant (p = 0.064, Kruskal-Wallis). Aggressive behaviors included biting, boxing, mounting, and chasing. Male methyl diet animals were, on average, more aggressive than control diet males, but this was statistically insignificant (p = 0.069). In both cases, bars represent standard error.

Female methyl diet animals performed significantly higher numbers of repetitive behaviors than control diet females (Fig. 4A; p<0.01, Kruskal-Wallis test). Examples are shown in Video S1. Female methyl diet animals were, on average, more social, but this was not deemed significant (Fig. 4B; p = 0.064, Kruskal-Wallis). Similarly, male methyl diet animals trended towards more aggression than control diet males, but this was not statistically significant (p = 0.069, Kruskal-Wallis test). A^Nb^ animals are more aggressive and exhibit less repetitive behavior than standard BW animals [20]. Thus, it is possible that some of these behavioral effects are due to suppression of the agouti (or a tightly linked) locus itself.

## Discussion

We set out to assess whether the methyl-donor diet would affect the *Peromyscus* natural agouti variant A^Nb^ in a similar manner to the *Mus* A^vy^ and whether the behavior of these wild-derived animals was obviously altered by the diet. The data presented here further indicate that these dietary components do indeed affect the A^Nb^ agouti allele, although whether this is via DNA methylation, or even a *cis*-effect, is unknown (our preliminary data does not suggest significant DNA methylation changes at the agouti promoter). The apparent lack of a retroelement at this allele suggests more broad effects than the mouse A^vy^ and axin^Fu^ studies. Further, female repetitive behavior and weights were significantly increased. Unexpectedly, the diet resulted in significant increases in mortality and abnormalities, with a greater effect in males.

The data presented here indicate that dietary intake of methyl-donors may have multiple adverse outcomes in a true wild-type mammalian model. To our knowledge, this is the first study to associate these particular defects, mortality or altered behavior in wild-type animals with these dietary factors.

We note that increasing evidence points to gene-environment interactions underlying the etiology of many diseases. Folic acid and other methyl-donor pathway components are typically thought of as preventing, rather than being causal to human health issues. Addition of these nutrients to flour appears to have dramatically reduced neural tube defects [1], and deficiencies are also thought to contribute to neuro-cognitive disorders [21]. However, this study adds to a growing number of recent studies suggesting deleterious effects of developmental exposure to high doses of these compounds [2], [22], [23], [24], [25], [26], [27], [28]. For example, mutations in some loci involved in neural tube development are exacerbated (rather than rescued) by excess folic acid [24], and neurons developmentally exposed to high folic acid may be more susceptible to seizure [26]. Further, studies using these same components have shown increased colitis susceptibility and allergic airway disease (e.g. allergic asthma) in standard laboratory mice (C57BL/6J) [29], [30].

Through counting of food pellets consumed, we estimated that these animals took in approximately one food pellet per day. This amount is roughly equivalent to a human consuming around 1750–2000 micrograms of folic acid in a day (based on weight of the animals and 0.0043 grams folate/kg food). We note that such consumption is quite feasible, as many commercial supplements contain 800 micrograms folate (e.g. http://www.vitaminshoppe.com/p/folic-acid-800-mcg-100-capsules/vs-1148#.UwetE8pWQ7w), which are taken in addition to the amounts found in enriched flour and sports drinks. Other ingredients in this diet are also consumed in copious amounts. For example, the decaffeinated version of the popular 5- hour energy drink contains additional Vitamin B12 and choline in addition to folic acid (http://www.5hourenergy.com/healthfacts.asp?Product=decaf). While rodent and human metabolism differ substantially, it is worth considering whether these dietary components may contribute to human behavioral variation [31].

Clearly, much additional work is required to assess the scope and mechanisms of these adverse effects. For example, we are currently undertaking additional behavioral assays (e.g. Barnes Maze). Besides molecular characterization of these changes, we plan to test the dietary effects on an interfertile species (*P. polionotus*), which is more social and less prone to repetitive behaviors [20]. We hypothesize that certain genotypes will be more susceptible to specific epimutations that result in neurological disorders or have other deleterious effects.

That is, we hypothesize that certain genotypes in combination with threshold amounts of these nutrients at specific developmental time points may result in negative effects. As observed in our studies, we predict that such effects will also be highly sexually dimorphic.

## Supporting Information

Video S1 Examples of repetitive behaviors in control and methyl-diet raised *Peromyscus maniculatus* during social interaction tests.
(WMV)
